# Immunity transfer in mule foals fed with good IgG quality colostrum

**DOI:** 10.1016/j.heliyon.2024.e25560

**Published:** 2024-01-30

**Authors:** Luca Turini, Bindi Francesca, Vernaccini Matilde, Bonelli Francesca, Meucci Valentina, Sgorbini Micaela

**Affiliations:** a Department of Agricultural, Food and Environmental Sciences, University of Pisa, Via Del Borghetto 80, 56124, Pisa, Italy; b Department of Veterinary Sciences, University of Pisa, Viale Delle Piagge 2, 56124, Pisa, Italy

**Keywords:** Mule foals, Serum IgG, Serum total protein, Radial immunodiffusion, Refractometer, Electrophoresis, Biuret

## Abstract

While the passive transfer of immunity in horse and donkey foals has been extensively studied, there is limited information for mule foals. Immunoglobulin type G (IgG) and serum total protein concentration (TP) were assessed at different sampling times to evaluate the correlation between serum radial immunodiffusion (SRID) with electrophoresis, refractometry, and dry chemistry analyzer (Biuret), and to estimate serum IgG concentrations using serum TP in mule foals. We analyzed a total of 30 samples collected at birth, and at 6, 12, 24, and 48 h of life from 6 mule foals by SRID, electrophoresis TP, biuret TP, and refractometry TP.

The SRID IgG concentration significantly increased from birth until T6 (p < 0.001). Serum TP analyzed with refractometry revealed differences between T0 and T12, T24 and T48 (p < 0.05), while a significant difference was observed with the biuret method between T0 and all the other sampling times (p < 0.001).

A strong correlation was found between IgG SRID and biuret TP (r = 0.69, p < 0.001), and a good correlation existed between IgG SRID, refractometry TP, and electrophoresis TP (r = 0.44, p < 0.01 and r = 0.39, p < 0.05, respectively).

All methods can be used to estimate the passive transfer of immunity in mule foals. TP refractometry and biuret TP values can be used to determine serum IgG concentrations in the blood of mule foals on their first day of life through the application of a specific equation.

## 1 Introduction

Mules are thought to be more resilient, cleverer, and less reactive than horses and donkeys, however, as equids, these species share several characteristics [1,2]. One of the most important for breeders and veterinarians is probably the extremely high need for colostrum that equid foals have ready after birth. The placentation of mules, donkeys and horses belongs to the diffuse epitheliochorial pattern, leading to almost no sharing between mare and foetus, including immunoglobulins. Equids type of placenta makes mule foal almost agammaglobulinemic at birth earning colostrum essential for foals' protection against infections during the neonatal period [3,4]. Quality of colostrum and timing of first meal is crucial for a good transfer of immunity between mare and foal, representing the first challenge a mule foals must face and a critical point in new-born management. Colostrum concentration of immunoglobulins type G (IgG), the main actor of foals’ transfer of immunity, decreases dramatically 24 h after delivery, thus foals need to ingest an enough colostrum to absorb a sufficient amount of colostral IgG in the very first hours of life [[5], [6], [7]]. Unsuccessful feed timing, colostrum quality or quantity will lead to the failure of the Transfer of Passive Immunity (FTP) exposing the foal to potential severe infectious illnesses such as septicaemia, pneumonia, omphalitis, and septic arthritis [[8], [9], [10]].

Based on data about horse foals, the transfer of immunity is considered complete when serum IgG concentrations are ≥800 mg/dl, while concentrations between 400 and 800 mg/dl are considered partial FTP. If serum IgG concentrations are <400 mg/dl, total FTP is diagnosed [[11], [12], [13], [14]]. The gold standard for the determination of IgG concentrations in body fluids is the Serum Radial Immunodiffusion (SRID) assay which has the disadvantage of being expensive, time-consuming and results are susceptible to human errors or commercial kits used [15,16]. More feasible tools for estimation of serum IgG concentrations in foals are quick tests, such as the SNAP test (IDEXX SNAP® Foal IgG Test - SNAP® Foal IgG) [17]. These tests are easy to perform, practical in the field and relatively cheap, however, they are semi-quantitative tests giving ranges of IgG concentrations (≥800 mg/dl, 400–800 mg/dl, or < 400 mg/dl) instead of the exact amount of immunoglobulins absorbed.

Less used tests for assessing the transfer of passive immunity are the serum electrophoresis (ETP) which is still rapid, and results are not affected by operators but its feasibility in field conditions is low [18,19]; and refractometry. Despite colostrum and serum evaluation with refractometry is probably the largest method used in dairy farms for colostrum quality and passive transfer of immunity assessment [20,21], its implementation in foals has been limited [11,22]. Passive transfer of immunity can also be indirectly evaluated counting the amount of serum total protein (TP) which are mainly composed by immunoglobulins around birth [19,22]. Serum TP can be assessed with a refractometry or with an automated chemistry analyzer; this technique is unaffected by plasma lipids (lipoproteins, cholesterol), glucose, and urea present in serum, but cannot be used in the field and is very expensive [22].

Despite the extensive knowledge available regarding the immunity status of horse and donkey foals [6,7,[10], [11], [12]], few studies have been carried out about mules, thus the current study's objectives were to measure serum IgG and TP concentrations in new-born mule foals throughout the first two days of life and to investigate the correlation between SRID and other diagnostic methods like electrophoresis, refractometry, and dry chemical analysis.

## 2 Materials and methods

### 2.1 Animals and management

The study population was represented by 7 mule foals, all born at the Veterinary Teaching Hospital, Department of Veterinary Medicine, University of Pisa during the foaling season 2020–2022. Delivery was supervised and the APGAR score was immediately assessed by an expert operator [4], while another operator collected a sample of colostrum and assessed its quality by a Brix refractometer. All dams produced a high-quality colostrum defined as > 23 % on the Brix scale, thus mule foals were led to nurse from their own dam within the first 3 h of life [6,7,23,24].

### 2.2 Inclusion criteria

Inclusion criteria for dams were: (1) treated against gastrointestinal parasites and vaccinated against equine influenza, tetanus, and equine herpes virus-1; (2) unassisted delivery. Inclusion criteria for mule foals were: (1) fed with good quality colostrum from the dam (>23 % Brix scale) [6,7,23,24], (2) APGAR score ≥7 measured 5 min after birth [4]; (3) had a complete transfer of immunity evaluated at 24 h of life with the Snap Foal IgG test [24].

### 2.3 Clinical and sampling procedures

For all the procedures, only manual restriction was needed. Mule foals were clinically assessed at birth (T0), then at 6 (T6), 12 (T12), 24 (T24), and 48 (T48) hours of life by a complete physical examination. Contextually, 10 mL of blood were drained from the jugular vein of each mule foal to measure TP and IgG concentrations. Blood collected was centrifuged (Legend RT, Sorvall; ThermoFisher Scientific Inc., Waltham, MA) at 1.565 g for 15 min after sampling (10-mL BD Vacutainer glass serum tube, silicone-coated; Becton Dickinson and Co., Franklin Lakes, NJ). The obtained serum was kept at −20 °C until the analysis.

### 2.4 Determination of serum IgG and total protein

Samples were evaluated in a single batch. In the absence of a mule-specific SRID test, determination of serum IgG was performed using a horse-specific SRID assay (Horse IgG IDRing (R) Test – IDBiotech, France). This was possible due to the clear homology between horses and donkeys IgG [25,26]. Equine immunoglobulin standards included with the kit were used to create a standard curve that was used to compare test results. Moreover, samples were diluted by 1/150 before IgG assessing as suggested by the manufacturer's instructions because concentrations of IgG in serum samples were too high for being evaluated without dilution.

Concerning methods other than SRID, serum TP were measured using a temperature-compensating digital refractometer (AR200; Reichert Analytical Instruments, Reichert Inc., Depew, NY), and a dry chemistry analyzer (SAT450, Assel, Italy) by the biuret assay method recommended by the manufacturer (hereafter, biuret). Finally, electrophoresis was performed with a fully automated electrophoresis instrument (Pretty Interlab, Sebia Company, Italy).

### 2.5 Statistic analysis

The minimum number of animals for inclusion in this study was calculated using G-power analysis with an effect size (Cohen coefficient) of 0.8, an alpha value of 0.05, and a power of 0.8. According to G-power analysis, the minimum number of animals required was four. The Shapiro-Wilk test was used to assess data for normal distribution.

The following linear model was used to assess data on the IgG SRID, TP Refractometry, TP Biuret, and ETP:

$$y_{\text{ijz}}=\mu +T_{i}+A_{j}+\varepsilon_{\text{ij}}$$

where y_ijz_ = dependent variables; T_i_ = fixed effect of the ith time of sampling (T0, T6, T12, T24 and T48); A_j_ = random effect of the jth animal (7 levels); ε_ij_ = random residual.

Treatment effects were considered significant at P < 0.05, and the reported least-square means and standard errors were provided.

Pearson's coefficient was used to assess correlations between IgG SRID, TP Refractometry, TP Biuret, and ETP; only correlations with a p-value of the linear model less than 0.01 were declared significant. The *t*-test with Tukey's correction was used to examine the linear contrasts in each parity level of the first model.

The whole statistical analysis was performed using JMP software (SAS Institute Inc., Cary, NC, United States).

## 3 Results

From the 7 mule foals enrolled at the beginning of the study, 6 met the inclusion criteria. They never exhibit signs of disease or discomfort during the time of the study. One (mule foal n. 7) was excluded because he showed failure of transfer of passive immunity. Considering 5 sampling times for each animal included, a total of 30 samples were analyzed.

Data distribution was normal, thus results about IgG and TP concentrations assessed with different methods have been reported as mean ± deviation standard (Table 1).

**Table 1:** Data from the evaluation of serum IgG and total protein concentrations assessed by different methods in 6 mule foals in the first 48 h of life (total of 30 samples) reported as mean and standard deviation.

Hours Post-partum	n	IgG SRID (g/dL)	TP Biuret (g/dL)	TP Refractometry (g/dL)	ETP (g/dL)
0	6	0.0 ± 0.0^A^	4.58 ± 0.50^A^	5.23 ± 1.03^A^	4.43 ± 0.58
6	6	1.66 ± 0.44^B^	5.70 ± 1.12^B^	5.72 ± 1.03^AB^	4.93 ± 1.15
12	6	2.10 ± 0.52^B^	6.22 ± 1.00^B^	6.16 ± 0.74^B^	5.31 ± 1.48
24	6	1.98 ± 0.62^B^	5.98 ± 0.87^B^	6.40 ± 1.12^B^	5.04 ± 1.27
48	6	2.31 ± 0.75^B^	5.51 ± 0.67^B^	6.40 ± 1.60^B^	5.46 ± 1.39
All time points	30	1.61 ± 0.98	5.59 ± 0.98	5.98 ± 1.15	5.04 ± 1.19
p-value		***	***	*	ns

Legend: IgG – immunoglobulin type G; SRID – serum radial immunodiffusion; TP – total protein; ETP – electrophoresis total protein. A≠B. * = p < 0.05, ** = p-value between 0.01 and 0.001; *** = p-value <0.001.

Table 2 showed results of the correlation analysis performed between each methods used to assess mule foals’ serum IgG and TP concentrations.

**Table 2:** Data about the coefficient of correlation values of serum IgG and total protein concentrations assessed by different methods in 6 mule foals.

	IgG SRID (g/dL)	TP Biuret (g/dL)	TP Refractometry (g/dL)	ETP (g/dL)
IgG SRID (g/dL)	1.00	*** 0.69	** 0.44	* 0.39
TP Biuret (g/dL)		1.00	* 0.38	* 0.41
TP Refractometry (g/dL)			1.00	^ns^ 0.25
ETP (g/dL)				1.00

Legend: IgG – immunoglobulin type G; SRID – serum radial immunodiffusion; TP – total protein; ETP – electrophoresis total protein; * = p < 0.05; ** = p-value between 0.01 and 0.001; *** = p-value <0.001, ns = not significant.

Serum TP Biuret and serum IgG SRID were shown to be significantly correlated. The serum IgG value was calculated using a serum TP Biuret with the following formula: *SRID IgG (g/dL)* = *-1.94 + 0.63 x TP Biuret value (g/dL).* The serum IgG SRID and serum TP Refractometry were shown to be significantly correlated. The value of serum IgG was calculated using a serum TP Refractometry with the following formula: *SRID IgG (g/dL)* = *-0.85 + 0.41 x TP Refractometry value (g/dL).* Serum IgG SRID and serum ETP did not significantly correlate.

Both formulas proposed in this study could be used by vets and breeders to estimate the amount of serum IgG using a different evaluation technique.

## 4 Discussion

To the best of our knowledge, only one study has investigated the passive transfer of immunity in mule foals and the various methods used to assess it [3].

For all time points, except for T0, the mean serum IgG concentration measured in this study using the SRID method was higher than the concentrations previously reported for foals [13,22] and donkey foals [6,19,27]. The mean serum TP concentration found in this study using a digital serum TP refractometer, was greater than what found in several other investigations [[11], [12], [13],^19^]. Discrepancies between IgG and TP concentration values found in the current study and others might be due to the different management strategies, animal diets, or breeds used [12,[28], [29], [30]].

The SRID IgG concentration increased significantly from birth until T6. Data from the present study showed that at birth (T0) mule foals seemed to be more agammaglobulinemic compared to foal and donkey foals [12,19]. This is an interesting result and highlights the importance of the correct transfer of passive immunity in these animals.

The dry chemistry analyzer revealed a significant difference between T0 and all the other sample intervals, as already reported for SRID analysis, whereas the refractometry analysis of serum TP showed a statistically significant difference between T0 and T12, T24, and T48. According to these results, in case of SRID or dry chemistry analyzer assessment, the first evaluation of passive immunity transfer could be done starting from 6 h after birth. Compared to donkeys [19] and horses [31,32], where serum IgG concentrations are generally performed around 12 and 12/18 h of life, respectively, it seems that passive transfer of immunity assessment can be done earlier in mules. This might enhance faster decision and intervention in case of need, improving the mule foals' chances of survival [17]. In the case of doubt, a second evaluation should be performed at 24 h as reported for other species.

Despite a trend of increased total protein as assessed by ETP over time, no statistically significant variations were seen across sample periods. The trend of our results agrees with literature confirming that the ETP concentration increased during the first hour of life in equine and donkey foals [19,32]. On the other hand, the lack of a statistically significant rise over time is unexpected [14]. Our population was composed by enough animals based on the power analysis results, however, further studies including an increased number of mule foals might clarify this result. The IgG SRID showed a good correlation with TP Biuret and TP Refractometry and no correlation with ETP.

The coefficient of correlation between IgG SRID and TP Refractometry was much lower in our mule foals compared to donkey foals [19] and equine foals [11,16]. Comparison between this result and literature is challenging because there are few studies that compared different methods for passive transfer of immunity assessment in equids and often statistical analyses and methodology vary a lot.

The correlation between IgG SRID and Biuret total protein in our mule foals was lower compared to donkey foals [19] and equine foals [18].

The results of this research indicate that several formulae may be utilized to estimate serum IgG when determining serum TP using the refractometry and dry chemical analyzer techniques. However, because of the significant dispersion around the regression curve, the plasma IgG concentration can only be predicted within a broad prediction interval. The immunoglobulin that predominates in equine colostrum is IgG, and the measurements of IgG concentrations in the serum of foals during the initial hours postpartum are used to calculate FTP [[33], [34], [35]].

Due to its practicality, affordability, ease of use, and lack of reliance on operator expertise or environmental factors, breeders typically choose measuring serum TP concentrations using a digital refractometer [36]. Owners or veterinarians may utilize the equations proposed by this study that can estimate serum IgG by serum TP analysis. This can be useful particularly when the gold standard technique for IgG concentrations detecting is not feasible. Even if the power analysis indicated that the study had a sufficient number of animals, more research including a larger sample size is required to determine precise cut-off values. Furthermore, concerning SRID results, it is essential to consider a slight range of results due to the different kits used when comparing different studies.

## 5 Conclusions

In conclusion, a high correlation between IgG SRID and Biuret TP and a good correlation between IgG SRID, Refractometry TP, and Electrophoresis TP was found. Our findings suggest that all the techniques might be applied to assess the immunity transmission in mule foals in their early days of life.

## Funding

This research received no external funding.

## Institutional review board statement

The Ethics Committee of the University of Pisa (Organismo Preposto per il Benessere Animale, OPBA) approved the study with protocol number 22/19.

## Data availability statement

Data are available on a public repository (10.5281/zenodo.10203843).

## CRediT authorship contribution statement

**Luca Turini:** Writing – original draft, Software, Formal analysis, Conceptualization. **Bindi Francesca:** Writing – original draft, Validation, Methodology, Data curation. **Vernaccini Matilde:** Validation, Methodology, Data curation. **Bonelli Francesca:** Writing – review & editing, Conceptualization. **Meucci Valentina:** Methodology. **Sgorbini Micaela:** Writing – review & editing, Validation, Conceptualization.

## Declaration of competing interest

The authors declare that they have no known competing financial interests or personal relationships that could have appeared to influence the work reported in this paper.
